# Adhesive Pili in UTI Pathogenesis and Drug Development

**DOI:** 10.3390/pathogens5010030

**Published:** 2016-03-15

**Authors:** Caitlin N. Spaulding, Scott J. Hultgren

**Affiliations:** 1 Department of Molecular Microbiology, Washington University School of Medicine, St. Louis, MO 63110, USA; cspaulding@wustl.edu; 2 Center for Women’s Infectious Disease Research, Washington University School of Medicine, St. Louis, MO 63110, USA

**Keywords:** UTI, rUTI, CAUTI, pili, UPEC, chaperone-usher pathway (CUP) pili, *Enterococcus*, vaccine, antibiotic-resistance

## Abstract

Urinary tract infections (UTIs) are one of the most common bacterial infections, affecting 150 million people each year worldwide. High recurrence rates and increasing antimicrobial resistance among uropathogens are making it imperative to develop alternative strategies for the treatment and prevention of this common infection. In this Review, we discuss how understanding the: (i) molecular and biophysical basis of host-pathogen interactions; (ii) consequences of the molecular cross-talk at the host pathogen interface in terms of disease progression; and (iii) pathophysiology of UTIs is leading to efforts to translate this knowledge into novel therapeutics to treat and prevent these infections.

## 1. Introduction

Urinary tract infections (UTIs) can be acquired in the community or hospital setting and are one of the most common bacterial infections that occur, affecting more than 150 million people worldwide each year [1,2,3]. UTI is clinically divided into two major infections, characterized by the localization of the bacteria in the urinary tract, cystitis and pyelonephritis. Cystitis, or lower UTI, is infection of the bladder. Once in the bladder, bacteria can ascend the ureters and colonize the kidneys, causing pyelonephritis or upper UTI. While the incidence of pyelonephritis is fairly low (~0.3%–0.6%) it is particularly dangerous as uncontrolled bacterial infection can spread to the bloodstream, causing sepsis (which occurs in ~2% of pyelonephritis cases) [4,5]. UTIs are also categorized as uncomplicated or complicated infections. Complicated UTI occurs in patients with: (i) functional or structural urinary tract abnormalities; (ii) renal failure; (iii) immunosuppression; (iv) pregnancy; and/or (v) foreign bodies, such as indwelling catheters, placed within their urinary tract [6,7,8]. Catheter-associated UTIs (CAUTI), make up 70%–80% of all complicated UTI and are the most common type of nosocomial infection [4,9]. CAUTI are of particular concern as they result in high morbidity, increased mortality and are the most common cause of secondary sepsis in hospital patients. While complicated UTI affects individuals of both genders, uncomplicated UTI primarily affects otherwise healthy women [10]. Pyelonephritis often occurs in healthy, non-pregnant women but can be categorized as a complicated UTI because of the potential of developing a blood stream infection. For women, the lifetime risk of developing an uncomplicated UTI approaches 60% [5]. Of these women who experience an initial UTI, 20%–30% will go on to experience a recurrent infection (rUTI) within 4–6 months, despite receiving appropriate antibiotic therapy [5,11]. To combat rUTI, these women are treated with frequent antibiotic therapy often to be taken at the time that symptoms arise or immediately following sexual intercourse [6]. However, a subset of these women will continue to experience rUTI as frequently as six or more times a year [12]. Due to its prevalence and high rates of recurrence, UTI is associated with significant economic costs. The financial burden of UTI in the United States, which reflects both direct medical costs and indirect costs such as lost work output and wages, is an estimated $5 billion annually [5]. These infections also result in significant patient morbidity resulting in serious deterioration in quality of life including: pain, discomfort, disruption of daily activities, and few treatment options other than long-term antibiotic prophylaxis [5,11,13].

**Figure 1:**
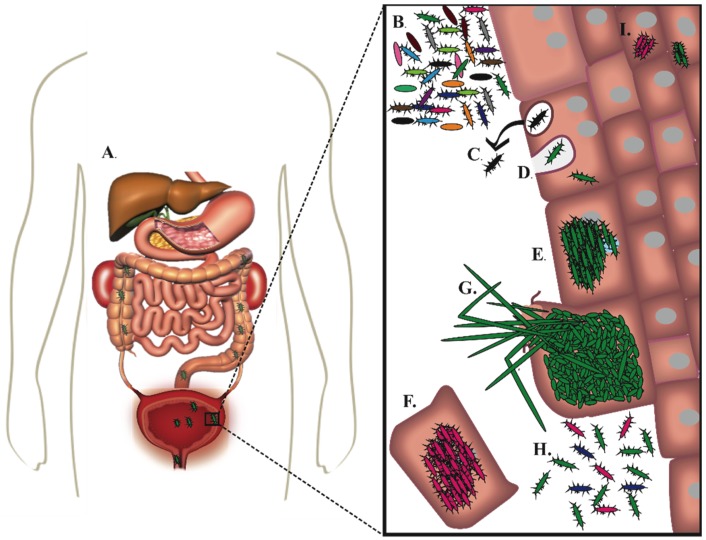
Uropathogenic *E. coli* (UPEC ) pathogenic cascade during cystitis. (**A**) UPEC residing in the gut are shed in the feces and colonize the peri-urethral and vaginal areas before ascending into the bladder. Upon accessing the bladder, UPEC adhere to the surface of superficial facet cells that line the bladder lumen in a type 1 pili dependent manner (**B**). Adherent bacteria invade into the facet cells and are either expelled back into the lumen by the cell in a TLR-4 dependent manner [19] (**C**) or escape from the endocytic vesicle into the cytoplasm (**D**). Upon invasion, bacteria replicate in the cytoplasm forming intracellular bacterial communities (IBCs) (**E**). One host mechanism of defense against intracellular UPEC is the shedding of urothelial cells into the urine (**F**), which reduces the overall number of UPEC in the bladder. During the late stages of IBC formation, filamentous bacteria dissociate from the IBC, burst out of the cell and back into the bladder lumen where they remain or can invade an adjacent facet cell (**G**). There are two potential outcomes of infection: chronic cystitis or resolution of infection. Uncontrolled bacterial replication in the urine occurs in mice that develop chronic cystitis (**H**). In mice that resolve infection, small pockets of bacteria, termed quiescent intracellular reservoirs (QIRs), form and reside in the underlying urothelium and may seed future rUTI (**I**).

While many bacterial organisms cause UTI, the most common causative agent of both uncomplicated and complicated UTI is the gram-negative pathogen uropathogenic *Escherichia coli* (*E. coli*) (UPEC). UPEC are responsible for 80%–90% of all uncomplicated UTI and approximately 65% of complicated UTIs [5,14,15]. Gram-positive *Enterococcus* species are the second leading cause of complicated UTI (11%) and the third leading cause of uncomplicated UTI (5%) [15]. The source population of UPEC and *Enterococcus* that lead to UTI is thought to be the gastrointestinal tract, where they can reside as either commensal or transient members of the gut microbiota [11,16,17]. When present in the gut, UPEC or *Enterococcus* spp. can be shed in the feces, inoculating peri-urethral or vaginal areas, and are subsequently introduced into the urinary tract during periods of physical manipulation such as during sexual activity or catheterization (Figure 1A) [18]. Upon entering the bladder, uropathogens must bind to an available epithelial receptor and/or, if present, abiotic-surface to establish and maintain colonization. UPEC and enterococcal species both accomplish this through the expression of distinctive adhesive pili on their surface. After creating a foothold in the bladder, uropathogens employ a myriad of additional virulence factors to establish bladder colonization in the face of an active immune response, micturition, and rapid epithelial cell exfoliation. Historically, antibiotics have been used, very successfully, to treat patients with UTI. However, the rise of single and multi-drug resistant uropathogens as well as high rates of recurrence in women infected with both antibiotic sensitive and drug-resistant uropathogens has become a major concern, highlighting the need to develop alternative strategies to treat patients with UTI and CAUTI. In this review, we will discuss the role of adhesive pili during UTI or CAUTI. Here we will focus mainly on UTI and CAUTI caused by UPEC and *Enterococcus spp*. due to the high prevalence of these pathogens in community-acquired and nosocomial infections. We will also explore the development of alternative, non-antibiotic treatment strategies that target adhesive pili in order to prevent UPEC and *Enterococcus* spp. from initiating infection and thus causing disease.

## 2. The Role of Chaperone-Usher Pathway (CUP) Pili in UPEC Mediated UTI

### 2.1. CUP Pilus Assembly Mechanisms

**Figure 2:**
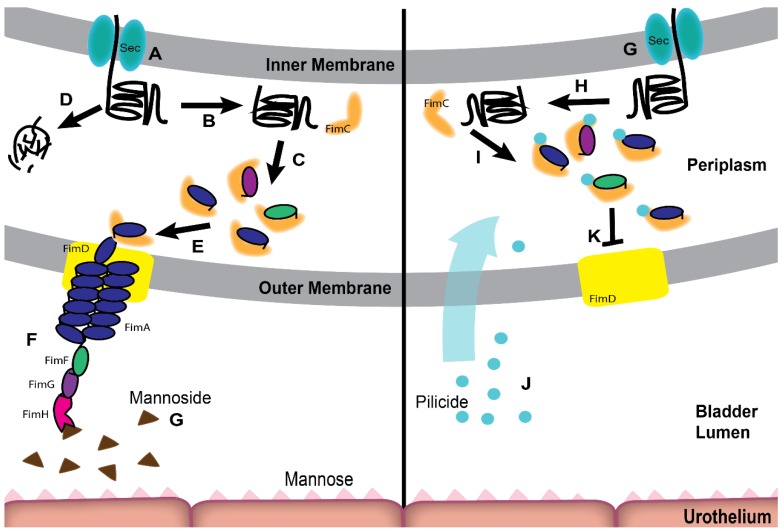
Mannosides and Pilicides prevent uropathogenic *E. coli* (UPEC) UTI by targeting the function or formation of type 1 pili. (**A,G**) Unfolded pilus subunits are secreted to the periplasm by the Sec apparatus. (**B,H**) Upon entering the periplasm, unfolded subunits immediately interact with the cognate chaperone (FimC). Subunits have an incomplete Ig-like fold which must be completed in order to properly fold. In a process called donor strand complementation (DSC) FimC donates its G1 β-stand to the subunit, stabilizing it (**C,I**). Subunits that do not interact with FimC are unable to fold correctly and are degraded (**D**). The chaperone then delivers the subunit to the outer member usher, FimD (**E**). Upon reaching FimD the subunit is assembled into the maturing pilus via donor stand exchange (DSE) with the adjacent pilus subunit (**F**). Mannosides prevent type 1 pilus function by binding, in an irreversible manner, to FimH and therefore prevent the interaction of FimH and mannose on the bladder surface (**G**). Pilicide works by halting pilus assembly. These molecules enter the periplasm (**J**) and bind to the pilus chaperone, halting assembly (**K**).

Upon entering the bladder, UPEC must first adhere to the bladder epithelium, also referred to as the urothelium, or risk clearance during urine voiding. Recognition and attachment to host and environmental surfaces is mediated through the expression of non-flagellar, adhesive, extracellular fibers, called pili that bind to receptors present on the host cell surface. In UPEC, many of these adhesive pili belong to a large, conserved family of pili called the chaperone-usher pathway (CUP) pili [20]. CUP pili are assembled by the corresponding chaperone-usher machinery, which are encoded by operons that contain all the dedicated genetic information necessary to assemble a mature pilus: an outer-membrane pore-forming usher protein, a periplasmic chaperone protein, pilus subunits, and in most cases, a tip adhesin protein. The first crystal structure of a CUP chaperone, PapD, which is involved in the assembly of P pili, revealed that it consists of two-immunoglobulin (Ig) domains [21]. Two key amino acid residues, R8 and K112, present in the cleft of the chaperone were subsequently identified as the active site of the protein [22]. Unlike the chaperone, pilus subunits are composed of an incomplete Ig fold, which lacks the C-terminal beta strand and requires the assistance of the dedicated chaperone for folding and stability (Figure 2B,D). Chaperone-assisted folding occurs by a reaction termed donor strand complementation (DSC) in which conserved alternating exposed hydrophobic residues on the chaperone’s G1 strand are buried in complementary pockets in the pilus subunit, allowing for the completion of the subunits Ig fold (Figure 2C) [23,24]. This interaction allows pilus subunits to fold into a primed, high-energy state in complex with the chaperone, ultimately allowing the subunits to be targeted to the outer membrane usher (Figure 2E). The usher, a gated channel, is made up of five functional domains: a 24 stranded transmembrane β-barrel translocation domain (TD), a β-sandwich plug (PLUG) that resides in the pore of the TD in the apo-usher, an N-terminal periplasmic domain (NTD) and two C-terminal periplasmic domains (CTD1 and 2) [25,26,27,28,29]. The usher catalyzes pilus assembly by driving subunit polymerization in a concerted reaction termed donor strand exchange (DSE) (Figure 2E,F) [30,31,32,33]. Pilus subunits, excluding the adhesin, encode an N-terminal extension (Nte) comprised of conserved alternating hydrophobic residues. DSE occurs when the chaperone is displaced and an incoming subunit’s Nte zips into the previously chaperone-bound groove of a nascently incorporated subunit at the growing terminus of the pilus. This “zip-in-zip-out mechanism” allows for the final folding of the pilus subunit, such that every subunit in the pilus completes the Ig fold of its neighbor (Figure 2F).

### 2.2. The Role of Type 1 Pili during Uncomplicated UTI

A hallmark of the luminal surface of urothelial cells, also called superficial facet cells, are the presence of uroplakin plaques, which consist of 4 uroplakin integral membrane proteins, that function as a barrier between the toxic contents of the bladder and the underlying urothelium [34,35]. The surface of the uroplakin is studded with mannose [36,37]. The tip adhesin for type 1 pili, FimH, binds mannose with stereochemical specificity. Therefore, upon entering the bladder, FimH is able to bind mannosylated residues on the bladder surface, such as those found on uroplakin as well as β1-α3 integrin receptors (Figure 1B) [36,38,39,40]. FimH mediated interaction with the urothelium induces a signaling cascade that activates Rho family GTPases and results in actin rearrangement within urothelial cells, promoting UPEC invasion (Figure 1C,D) [38,41,42]. Studies in mice have revealed that once inside superficial facet cells, UPEC are able to escape the endocytic vesicle, via unknown mechanisms, into the cytoplasm where they replicate rapidly forming biofilm-like communities called intracellular bacterial communities (IBCs) (Figure 1D,E) [43,44,45]. In mice, type 1 pili are not only required for cellular adherence and invasion but also for the aggregation of bacteria into an IBC in the host cell cytoplasm [46]. Mouse studies have shown that invasion is a critical step in UPEC pathogenesis in naïve mice, allowing the bacteria to rapidly replicate in a niche protected from many innate immune defense mechanisms and antibiotics. UPEC that cannot invade the urothelium, like those lacking FimH, are quickly cleared from the bladder, emphasizing the importance of this intracellular stage to the success of the pathogen [46]. Mature IBCs contain approximately 10^4^ bacteria. Upon maturation, bacteria within the IBC adopt a filamentous morphology and disperse from the biomass, fluxing out of the cell and back into the bladder lumen where they can adhere to and/or invade adjacent facet cells or exposed transitional epithelial cells (Figure 1G,H) [43,44,47,48]. Importantly, evidence of IBCs and bacterial filaments have been observed in women suffering from acute UTI, one to two days post self-reported sexual intercourse, but not in healthy controls or infections caused by Gram-positive organisms, which do not form IBCs [49]. IBCs have also been observed in urine from children with an acute UTI and their presence was correlated with recurrent UTI, supporting the validity of their importance in pathogenesis and the ability of the mouse model to recapitulate human disease [50].

While the collection of tissue biopsies from women with UTI is generally contraindicated, one study of tissue biopsies demonstrated the presence of intracellular UPEC [51]. In urine from UTI patients and/or mice, the expression of type 1 pili has been reported to be variable [52,53,54]. Several studies, in mice and humans, have demonstrated that UPEC attached to shed epithelial cells in the urine express type 1 pili while planktonic UPEC found in the urine tend to be nonpiliated [54,55]. This suggests that the expression of type 1 pili is niche specific, with type 1 pili being expressed by UPEC that are attached to the epithelial surface. 

In addition to type 1 pili, a mosaic of UPEC virulence factors including adhesins, toxins, capsule, siderophores, and flagella have been identified and characterized [39,56,57,58,59,60,61]; however the direct contribution of these putative urovirulence factors has not been exhaustively studied. In a recent comprehensive genomic analysis of 43 UPEC isolates, we found that “core” genes, defined as genes shared between all *E. coli*, constituted approximately 60% of each strain’s genome. Further, we were unable to find any single set of genes that accurately delineated UPEC from non-UPEC strains (Schreiber and Hultgren, personal communication). The lack of a strict ‘genetic’ definition of UPEC indicates that, in most cases, urovirulence in UPEC is more complicated than carriage of a set of virulence-associated genes that are missing in non-UPEC strains. Instead urovirulence in UPEC strains may be defined by alternative characteristics, like propensity to certain patterns of gene expression, as is seen with niche specific expression of type 1 pili in the bladder [54,55], or the carriage of different alleles of core genes. There are a number of different alleles of FimH, described later in this review, that alter the affinity of the adhesin for its ligand and may be associated with more virulent strains. These alternative characteristics may explain how genes that are highly conserved in both UPEC and non-UPEC strains, like those that encode type 1 pili, contribute to pathogenesis during UTI.

### 2.3. The Host Response to Type 1 Piliated UPEC

The host has evolved several mechanisms to respond to type 1 pilus mediated binding in a manner that favors bacterial clearance or killing. Caspase-dependent apoptosis and exfoliation of superficial facet cells is observed within hours of type 1 pili binding and invasion in C57BL/6 mice [39,62,63]. This promotes the clearance of infected cells from the host during micturition (Figure 1F). In the absence of cell exfoliation, the host can expel UPEC directly from an invaded cell via a TLR-4 dependent mechanism, reducing the number of successful UPEC invasion events and decreasing the overall number of IBCs formed during acute infection (Figure 1C) [19]. UPEC interactions with the host also leads to the robust recruitment of immune cells, mainly neutrophils and inflammatory monocytes, to the urinary tract as well as the up regulation of pro-inflammatory cytokines which drive the mobilization of additional immune effectors [64,65,66,67,68].

### 2.4. The Biophysics of FimH Structure and Function

Several natural allele variants of FimH exist. The residues in the mannose-binding pocket of FimH are invariant in all sequenced UPEC isolates [37,69], however there is diversity outside of this pocket, including at a cluster of amino acids that are positively-selected in UPEC strains [69]. Interestingly, mutations in these positively selected residues results in distinct FimH alleles that are skewed into either elongated or compact conformations, which have been observed in several X-ray crystal structures of FimH [25,26,70]. The elongated conformation corresponds to the mannose binding conformation whereas the compact form binds mannose only weakly or not at all [69,71,72]. When FimH is bound to the FimC chaperone, it adopts an elongated conformation [26] however, upon its incorporation into the tip of the pilus, it can convert to a compact conformation with an altered mannose-binding pocket [25,70]. In the FimCH complex, all of the FimH allele variants tested have a high affinity for mannose. However, when incorporated into a tip-like state, following a DSE reaction with the FimG adaptor, FimH variants differ greatly in their mannose affinity suggesting that depending on the allele, FimH is skewed toward either an elongated or compact form after its incorporation into the tip [73]. Interestingly, UPEC strains containing distinct FimH alleles also display altered levels of pathogenicity during acute and chronic UTI in C57BL/6 [74] and C3H/HeN mice [73]. Of particular interest we found that specific mutations in two positively selected residues, A27 and V163, result in a FimH variant (FimH(A27V/V163A)) that resides within a tip-like complex predominantly in a high-affinity mannose binding conformation [73]. Surprisingly, this FimH allele is severely attenuated in a cystitis model [69,73]. These findings support the hypothesis that FimH resides in an equilibrium of mannose-binding and non-binding conformations, which are governed, in part, by the identity of select residues that are under positive selection in UPEC isolates. The inter-conversion between high and low mannose-binding conformations is likely important for tissue-binding and/or immune evasion, which may begin to explain why the FimH(A27V/V163A) variant, which appears to be “locked” in a high-affinity elongated state is unable to cause infection *in vivo* despite its high-affinity for mannose. These findings may be related to the important observation made by others that FimH conformation is influenced by shear force leading to a “catch bond” model of FimH function [70,71,72,75,76]. Thus, the ability of FimH to be incorporated into a pilus tip to interconvert into different conformations affects virulence.

### 2.5. The Role of P Pili in Pyelonephritis

The ability of UPEC to ascend from the bladder to the upper urinary tract likely involves many genetic and environmental factors, one of which is thought to be the up-regulation of P pili and corresponding down-regulation of type 1 pili [77]. P pili, which are encoded by the *pap* operon, mediate adhesion of UPEC to kidney tissue resulting in pyelonephritis [61,78,79,80]. This binding is dependent on the expression of the P pilus tip adhesin, PapG [56,81]. Three alleles of PapG exist (PapG-I, -II, and -III) and each allele shows a distinct affinity for a series of Galα1-4Gal containing glycolipid receptors, which are expressed to various degrees in the kidneys and ureters of mammals [82]. Human kidneys, for example, abundantly express the ligands for PapG-II, globoside, and thus are colonized with UPEC that express PapG-II alleles. Conversely, PapG-III binds strongly to Forssman glycolipid, which is present in dog but not in human kidneys. Thus, different alleles of PapG mediate host tropisms [83,84,85].

### 2.6. Identifying Roles for Additional CUP Pili Types during Disease

A recent analysis of 35 *Escherichia* spp. genomes and 132 plasmids identified a total of 458 CUP pili operons, representing 38 distinct CUP pilus types based on usher phylogeny [86,87]. CUP pili tipped with specific adhesins provides *E. coli* the ability to bind to distinct ligands with stereo-chemical specificity. Single UPEC strains were found to carry as many as 16 distinct, intact CUP pilus systems that likely aid UPEC strains in their colonization of many host niches, including the gut, vagina, urethra, bladder, and kidneys. Thus, it is likely that the retention of many distinct CUP pilus types reflects the adaptation to broad environmental and host niches and their function in facilitating the various tropisms of *E. coli* [88]. Aside from type 1 and P pili, UPEC strains encode a number of additional CUP pilus types for which a role in UPEC pathogenesis is minimally defined or completely undetermined. S pili are known to bind sialosyloligosaccaride residues in host cells and likely mediate infection of the ureters and kidneys [89]. Clinical isolates containing S pili are often associated with more severe outcomes in patients with UTI, including: pyelonephritis, sepsis, and meningitis. The Dr pili adhesin has been shown to bind type IV collagen and decay-accelerating factor (DAF), both of which are expressed in human kidneys. Dr pili enable UPEC colonization of the mouse renal interstitial basement membrane [90,91]. The roles for CUP pili, including F1C, F9, Ygi, Yad, and Auf, have been briefly explored and have been recently reviewed [92,93,94]. Identifying roles for additional CUP pilus types will enhance our overall knowledge of UTI pathogenesis and may provide novel drug targets to treat patients with UTI.

### 2.7. Outcomes of UTI: Connecting Findings in Humans and Mice

Much of our understanding of UTI and rUTI pathogenesis comes from studies in mice. The development of murine models of acute and recurrent cystitis have allowed for many important advances in our understanding of UTI pathogenesis. However, it is also worth noting that the UTI mouse models, like all animal models, have limitations. Behavior, genetic, and environment differences between mice and humans make it challenging to translate all murine findings to humans [95]. Here we discuss advances in our understanding of UTI pathogenesis based on experiments performed in relevant mouse models and highlight how these findings have been recapitulated in clinical studies performed in women with UTI or rUTI.

For women with UTI, several disease outcomes are possible, including: asymptomatic bacteruria (ABU), acute self-limiting infection, or chronic/recurrent UTI [5]. Placebo studies in women have shown that approximately 50% of UTIs do not resolve in the absence of effective antibiotic treatment, implying that cystitis is not always self-limiting. These findings highlight the importance of clinical intervention to resolve UTI and the developing crisis of emerging multi-drug resistant uropathogens [96,97]. In naïve mice, there are two major outcomes of UTI: (i) self-limiting acute infection that resolves within days of the initiation of infection with or without the formation of quiescent intracellular reservoirs (QIR); or (ii) persistent, high-titer bacteruria concomitant with high bladder bacterial burden and severe bladder inflammation which, in the absence of antibiotic intervention, can last for the lifetime of the animal and is referred to as chronic cystitis [98] (Figure 1H). The resolution of infection in mice is often accompanied by QIR formation [99] (Figure 1I). The exfoliation response results in the exposure of the underlying transitional cells, which allows the subsequent invasion of UPEC into these cells. However, unlike superficial facet cells, the underlying transitional cells do not support IBC formation; instead UPEC establish QIRs, which are comprised of 8–12 dormant bacteria in Lamp1+ vesicles [99]. The mechanism of QIR formation is unknown, however, these reservoirs are able to reactivate and release UPEC back into the bladder lumen to initiate a new infection cycle [99,100]. Interestingly, the fate of disease in mice is determined by whether an acute host-pathogen checkpoint, which is influenced by the genetic background of the mice, is triggered. C57BL/6 mice are resistant to chronic cystitis after a single infection; however, they can develop persistent bacteriuria and chronic cystitis when “superinfected”, by multiple transurethral inoculations within a 24 hour period [74]. Elevated levels of interleukin-6 (IL-6), keratinocyte cytokine (KC/CXCL1), and granulocyte colony-stimulating factor (G-CSF) in the serum of C57BL/6 mice prior to the second infection predicted the development of chronic cystitis [74]. These same cytokines have been found to precede chronic cystitis in singly infected C3H/HeN mice, which are prone to the development of chronic cystitis [98]. Superinfection of C3H/HeN mice within a six-hour period doubles the proportion of mice that developed chronic cystitis. Intracellular bacterial replication, regulated hemolysin (HlyA) expression, and caspase 1/11 activation were essential for this increase [74]. The chronic bladder inflammation that accompanies chronic cystitis includes lymphonodular hyperplasia in the bladder submucosa and urothelial hyperplasia that results in the loss of superficial facet cells. Similar histological findings have been observed in humans suffering from persistent bacteriuria and rUTI [101,102].

Proteomic analysis of chronically infected bladders indicates that chronic inflammation also results in bladder epithelial remodeling, which may help to explain why mice that experience chronic cystitis are more susceptible to rUTI upon further bacterial challenge weeks after antibiotic intervention and resolution of the initial infection [67]. Studies of mice with rUTI may also begin to explain why a history of UTI is a major risk factor for rUTI in women, highlighting the clinical relevance of this mouse model. Elevated soluble serum biomarkers that were predictive of rUTI were also detected in young women with UTI [67]. Interestingly, temperance of the neutrophil response during the first 24 h of infection, by inhibition of cyclooxygenase-2 (COX 2), protected mice from chronic and recurrent cystitis [67]. These findings may help to explain the outcome of a small clinical trial that compared the outcomes of women with UPEC UTI after being given a three-day course of either ciprofloxacin or ibuprofen. The study found no difference in UTI outcome between these two groups at four or seven days post treatment [103]. Another study found that symptomatically treating women suffering from uncomplicated UTI with ibuprofen (without any antibiotics) resolved infection in two-thirds of the study group [104]. These results indicate that ibuprofen, which works by inhibiting COX 1 and 2, may act to decrease the severity of UTI in these patients. However, the second study also found that women in the ibuprofen treated group suffered from more UTI symptoms (abdominal pain, increased frequency of urination, dysuria) and were more likely to develop pyelonephritis [104].

The development of rUTI is likely a balance between bacterial factors and host genetics. Murine studies have indicated that the innate immune response is critical for combating UTI, which had been recapitulated in human clinical studies [105]. Therefore, mutations in the genes involved in this early immune response can greatly influence the susceptibility of a host to UTI. For example, certain polymorphisms and altered expression levels of innate immune genes, like Toll-like receptor 4 (TLR4) and CXCR1, have been associated with less severe symptomatic UTI but an increase in the development of ABU in women [105]. Alterations in the sequence or expression of other innate immune genes, like IRF3 and CXCR1, have been associated with increased incidences of acute pyelonephritis [105]. 

## 3. Pili are Critical for the Establishment of UPEC and *Enterococcus* Mediated CAUTI

The introduction of a catheter into the urinary tract provides an additional surface on which bacteria can adhere and establish infection. Uropathogens that are commonly associated with CAUTI, like those belonging to the *Enterococcus* genus, also encode a number of distinct adhesive factors and pili that permit attachment within the host. Of these factors, the endocarditis- and biofilm-associated (Ebp) pilus has recently been identified to play an essential role in the establishment and persistence of UTI in a catheterized mouse model [106,107,108,109,110]. Upon catheterization, implanted catheters are coated with host-derived fibrinogen and are subsequently bound by Ebp expressing *Enterococcus faecalis* (*E. faecalis*), a common enterococcal uropathogen [109]. The Ebp pilus binds fibrinogen via its tip adhesin, EbpA, which contains an N-terminal fibrinogen-binding domain [109]. The mechanism(s) by which fibrinogen enters the bladder is still under investigation but is likely due to bladder damage that occurs during the catheterization process and subsequent infection. Mechanical stress induced by insertion of the catheter into the bladder results in the induction of a robust inflammatory response and severe bladder edema in humans and mice [107,111,112,113]. Bladder inflammation, including increases in serum cytokines IL-1α and IL-6, and edema, is elevated further upon introduction of *E. faecalis* into the catheterized mouse [107]. Increases in IL-1α and IL-6 have been previously shown to stimulate the release of fibrinogen from the liver into the bloodstream as a part of the pro-inflammatory response and circulating fibrinogen may then leak into the urinary tract via tissue damaged during catheterization. Once in the bladder, fibrinogen is deposited on the catheter, providing a binding site for EbpA expressing *E. faecalis*. Catheterization and the subsequent inflammatory response are essential in mediating CAUTI, as mice infected with *E. faecalis* in the absence of a catheter are quickly cleared from the bladder [107]. Fibrinogen deposited onto the catheter surface not only enhances *E. faecalis* biofilm formation but also promotes growth, increasing the severity of the infection [109]. The ability to bind and utilize fibrinogen may be a general feature of infection in gram-positive bacteria, (as has been shown for *Staphylococcus* *aureus* (*S. aureus*)*, Staphylococcus epidermidis* (*S. epidermidis*), and Group A streptococci) and potentially in some fungal pathogens (as was recently shown for *Candida albicans*) [114,115]. The Ebp pilus also plays a role in the transition of *E. faecalis* infection from the bladder to the kidneys. In *E. faecalis*, loss of the pilus reduces bacterial colonization of the kidneys and thus lowers the incidence of pyelonephritis in infected mice [110].

UPEC, the leading cause of complicated UTI, can also use CUP pili to colonize the bladder during catheterization [15,116]. However, unlike *Enterococcal* spp., UPEC does not rely on the presence of the catheter to propagate high levels of bacterial colonization. In a CAUTI mouse model, UPEC undergoes the same acute pathogenic lifecycle that is observed in cystitis models, including: invasion into the tissue, IBC formation and maturation, the formation of filaments, and the emergence of filamentous bacteria back into the bladder lumen [116]. However, in the presence of the catheter, UPEC does appear to have a more robust extracellular population during acute infection, which is likely due to UPEC colonization and subsequent biofilm formation on the surface of the catheter [116]. Interestingly, type 1 pili are required for biofilm formation and UPEC colonization of the catheter [116]. These studies continue to define the importance of type 1 pili as a virulence factor during uncomplicated and complicated UTI in mice. 

## 4. Development of Pili-Based Alternative Treatments to Treat or Prevent UTI and CAUTI

Pili are potential drug targets due to the critical roles they play in UTI and CAUTI pathogenesis. Our understanding of the molecular mechanisms by which pili function and impact disease has permitted the development of novel therapeutics, such as vaccines and small molecule inhibitors, that target pili and block infection by preventing colonization. While a role for pili during intestinal colonization by uropathogens is currently unstudied, identifying and targeting pili that do permit intestinal colonization would provide a unique opportunity to target the source of uropathogens that lead to downstream infections.

### 4.1. Vaccines

A number of promising vaccines that target a wide-range of UPEC virulence factors are currently in development [117,118,119,120,121]. The FimCH vaccine, a subunit vaccine targeting the type 1 pilus adhesin FimH, is one such example. Significant protection was observed in subcutaneously vaccinated mice and cynomolgus monkeys given an intra-muscular vaccination, with the protected animals developing antigen-specific, long-lasting serum IgG antibodies [120,121]. Based on this data, it is presumed that the vaccine works by stimulating these FimH-specific IgG antibodies that block UPEC colonization of the bladder. Consistent with this, the polyclonal antibodies derived from vaccinated mice block FimH function and likely activate immune effector cells, like phagocytes and complement, for clearance of the infection [120,121]. The success of this vaccine in animal models highlights its potential as an alternative treatment to UTI in humans. A FimCH vaccine adjuvanted with PHAD (Avanti Polar Lipids, Alabaster, AL) is currently completing a Phase 1 human study. This study has enrolled about 67 women with and without a history of rUTI (personal communication, Gary Eldridge).

Other CUP pilus types are the focus of additional vaccine candidates. One such candidate is the PapDG subunit vaccine. P pili are strongly associated with pyelonephritis and as such have been designed to target UPEC and prevent complicated upper UTIs [61]. Intraperitoneal administration of the PapDG vaccine protects animals from infection and elicits a specific IgG antibody response in cynomolgus monkeys [118,122]. The Dr fimbriae represents another current vaccine target. Mice vaccinated with purified Dr fimbriae produce high titers of serum antibodies against the fimbriae but do not have lower colonization rates in either the bladder or kidneys [123]. 

Recent findings revealed an essential role for the Ebp pilus and its adhesin, EbpA, during *E. faecalis* mediated CAUTI in mice. Interestingly, vaccinating mice with EbpA results in significant protection against subsequent *E. faecalis* CAUTI compared to unvaccinated mice or mice vaccinated with other Ebp pilus protein subunits [109]. Vaccinating mice with only the N-terminal portion of EbpA, which contains the fibrinogen-binding domain, is also sufficient to protect mice from subsequent infection and may provide a higher level of protection than vaccination with the entire adhesin.

### 4.2. Small Molecule Inhibitors

The importance of the FimH-mannose interaction during infection in mice has prompted studies that test the effects of oral treatment with D-mannose or mannose-analogs on UTI outcome. Oral D-mannose treatment has been shown in studies to work as well as antibiotics to prevent rUTI. One group found that women who received prophylaxis with D-mannose after an initial UTI had similar rates of rUTI as a group that received antibiotic prophylaxis, indicating that D-mannose may be useful to prevent rUTI [124]. Another strategy to prevent UPEC pathogenesis has been the design of orally active, synthetic, small-molecule inhibitors of CUP pilus assembly or function. One such class of small molecule inhibitors are mannosides. Mannosides are mannose analogs that were rationally designed to bind within the mannose-binding pocket of FimH with a high affinity and thus block pilus binding of FimH to host receptors (Figure 2) [125,126]. Studies in mouse models have demonstrated that mannosides are potent, fast acting and highly efficacious in the treatment of UTI and CAUTI [116,127,128,129], highlighting their potential as a novel therapeutic strategy for UTI. Mannoside treatment is especially promising as a novel antibiotic sparing therapeutic because they are effective against multi-drug resistant uropathogens [128]. While D-mannose and mannosides both appear to effectively block FimH-mannose interactions, mannosides have approximately a 1,000,000-fold increase in potency for inhibiting FimH, making them promising antibiotic-sparing therapeutics [125,130]. 

Another class of compounds are pilicides, which are small, rationally designed 2-pyridinones that block pilus assembly (Figure 2) [131,132]. Pilicides block assembly by binding to the pilus chaperone, preventing chaperone-pilus subunit-usher interactions that are fundamental for pilus biogenesis [133]. While pilicides were originally designed to target type 1 pilus assembly, recent studies have found that pilicide treatment disrupts assembly of at least four-CUP pilus types (type 1-, P-, S-, and Dr- pili) as well as flagellar motility [134,135]. The ability of pilicides to target multiple pilus types makes it an extremely valuable treatment option. 

## 5. Conclusions

Complicated and uncomplicated UTI are extremely common, affecting a large portion of the global population. The high rate of infection and recurrence of UTIs, their monetary cost, their physical burden, and the increasing occurrence of antibiotic resistant uropathogens emphasize the need for effective treatments to combat the common causative agents of UTI and CAUTI, like UPEC and *Enterococcus* strains. Understanding the molecular mechanisms by which uropathogens are able to cause disease is the first step to identifying novel drug targets. Adhesive pili, like type 1, P, and Ebp, have been shown to play essential roles in UTI pathogenesis in mouse models and provide potential drug targets that may greatly reduce or prevent infection in patients. While the mouse models of uncomplicated UTI and CAUTI have been shown to mimic disease in human patients, the success of vaccine and small molecular inhibitor therapies against these pili in humans is dependent on determining the efficacy of these treatments in clinical trials.

## References

[1] Nicolle L.E. (2005). Catheter-related urinary tract infection. Drugs Aging.

[2] Stamm W.E., Norrby S.R. (2001). Urinary tract infections: disease panorama and challenges. J. Infect. Dis..

[3] Harding G.K., Ronald A.R. (1994). The management of urinary tract infections: what we have learned in the past decade. Int. J. Antimicrob. Agents.

[4] Foxman B. (2010). The epidemiology of urinary tract infection. Nat. Rev. Urol..

[5] Foxman B. (2014). Urinary tract infection syndromes: Occurrence, recurrence, bacteriology, risk factors, and disease burden. Infect. Dis. Clin. N. Am..

[6] Hooton T.M. (2012). Uncomplicated urinary tract infection. N. Engl. J. Med..

[7] Lichtenberger P., Hooton T.M. (2008). Complicated urinary tract infections. Curr. Infect. Dis. Rep..

[8] Levison M.E., Kaye D. (2013). Treatment of complicated urinary tract infections with an emphasis on drug-resistant gram-negative uropathogens. Curr. Infect. Dis. Rep..

[9] Lo E., Nicolle L.E., Coffin S.E., Gould C., Maragakis L.L., Meddings J., Pegues D.A., Pettis A.M., Saint S., Yokoe D.S. (2014). Strategies to prevent catheter-associated urinary tract infections in acute care hospitals: 2014 update. Infect. Control Hosp. Epidemiol..

[10] Foxman B., Brown P. (2003). Epidemiology of urinary tract infections: Transmission and risk factors, incidence, and costs. Infect. Dis. Clin. N. Am..

[11] Foxman B. (2002). Epidemiology of urinary tract infections: Incidence, morbidity, and economic costs. Am. J. Med..

[12] Ikaheimo R., Siitonen A., Heiskanen T., Karkkainen U., Kuosmanen P., Lipponen P., Makela P.H. (1996). Recurrence of urinary tract infection in a primary care setting: Analysis of a 1-year follow-up of 179 women. Clin. Infect. Dis..

[13] Al-Badr A., Al-Shaikh G. (2013). Recurrent Urinary Tract Infections Management in Women: A review. Sultan Qaboos Univ. Med. J..

[14] Griebling T.L., Litwin M.S., Saigal C.S. (2007). Urinary tract infection in women. Urologic Diseases in Amerca.

[15] Flores-Mireles A.L., Walker J.N., Caparon M., Hultgren S.J. (2015). Urinary tract infections: epidemiology, mechanims of infection and treatment options. Nat. Rev. Microbiol..

[16] Chen S.L., Wu M., Henderson J.P., Hooton T.M., Hibbing M.E., Hultgren S.J., Gordon J.I. (2013). Genomic diversity and fitness of *E. coli* strains recovered from the intestinal and urinary tracts of women with recurrent urinary tract infection. Sci. Transl. Med..

[17] Moreno E., Andreu A., Pigrau C., Kuskowski M.A., Johnson J.R., Prats G. (2008). Relationship between *Escherichia coli* strains causing acute cystitis in women and the fecal *E. coli* population of the host. J. Clin. Microbiol..

[18] Scholes D., Hooton T.M., Roberts P.L., Stapleton A.E., Gupta K., Stamm W.E. (2000). Risk factors for recurrent urinary tract infection in young women. J. Infect. Dis..

[19] Song J., Li B., Grady R., Stapleton A., Abraham S. (2009). TLR4-mediated expulsion of bacteria from infected bladder epithelial cells. Proc. Natl. Acad. Soc. USA.

[20] Busch A., Waksman G. (2012). Chaperone-usher pathways: Diversity and pilus assembly mechanism. Philos. Trans. R. Soc. Lond. B Biol. Sci..

[21] Holmgren A., Brändén C. (1989). Crystal structure of chaperone protein PapD reveals an immunoglobulin fold. Nature.

[22] Slonim L.N., Pinkner J.S., Branden C.I., Hultgren S.J. (1992). Interactive surface in the PapD chaperone cleft is conserved in pilus chaperone superfamily and essential in subunit recognition and assembly. EMBO.

[23] Sauer F.G., Futterer K., Pinkner J.S., Dodson K.W., Hultgren S.J., Waksman G. (1999). Structural basis of chaperone function and pilus biogenesis. Science.

[24] Barnhart M.M., Pinkner J.S., Soto G.E., Sauer F.G., Langermann S., Waksman G., Frieden C., Hultgren S.J. (2000). PapD-like chaperones provide the missing information for folding of pilin proteins. Proc. Natl. Acad. Soc. USA.

[25] Geibel S., Procko E., Hultgren S.J., Baker D., Waksman G. (2013). Structural and energetic basis of folded-protein transport by the FimD usher. Nature.

[26] Phan G., Remaut H., Wang T., Allen W.J., Pirker K.F., Lebedev A., Henderson N.S., Geibel S., Volkan E., Yan J. (2011). Crystal structure of the FimD usher bound to its cognate FimC-FimH substrate. Nature.

[27] Remaut H., Tang C., Henderson N.S., Pinkner J.S., Wang T., Hultgren S.J., Thanassi D.G., Waksman G., Li H. (2008). Fiber formation across the bacterial outer membrane by the chaperone/usher pathway. Cell.

[28] Thanassi D.G., Stathopoulos C., Dodson K., Geiger D., Hultgren S.J. (2002). Bacterial outer membrane ushers contain distinct targeting and assembly domains for pilus biogenesis. Bacteriology.

[29] Nishiyama M., Vetsch M., Puorger C., Jelesarov I., Glockshuber R. (2003). Identification and characterization of the chaperone-subunit complex-binding domain from the type 1 pilus assembly platform FimD. Mol. Biol..

[30] Volkan E., Kalas V., Pinkner J.S., Dodson K.W., Henderson N.S., Pham T., Waksman G., Delcour A.H., Thanassi D.G., Hultgren S.J. (2013). Molecular basis of usher pore gating in *Escherichia coli* pilus biogenesis. Proc. Natl. Acad. Soc. USA.

[31] Sauer F.G., Pinkner J.S., Waksman G., Hultgren S.J. (2002). Chaperone priming of pilus subunits facilitates a topological transition that drives fiber formation. Cell.

[32] Barnhart M.M., Sauer F.G., Pinkner J.S., Hultgren S.J. (2003). Chaperone-subunit-usher interactions required for donor strand exchange during bacterial pilus assembly. Bacteriology.

[33] Remaut H., Rose R.J., Hannan T.J., Hultgren S.J., Radford S.E., Ashcroft A.E., Waksman G. (2006). Donor-strand exchange in chaperone-assisted pilus assembly proceeds through a concerted beta strand displacement mechanism. Mol. Cell.

[34] Wu X.R., Sun T.T., Medina J.J. (1996). *In vitro* binding of type 1-fimbriated *Escherichia coli* to uroplakins Ia and Ib: Relation to urinary tract infections. Proc. Natl. Acad. Soc. USA.

[35] Sun T.T., Zhao H., Provet J., Aebi U., Wu X.R. (1996). Formation of asymmetric unit membrane during urothelial differentiation. Mol. Biol. Rep..

[36] Zhou G., Mo W.J., Sebbel P., Min G., Neubert T.A., Glockshuber R., Wu X.R., Sun T.T., Kong X.P. (2001). Uroplakin Ia is the urothelial receptor for uropathogenic *Escherichia coli*: Evidence from *in vitro* FimH binding. J. Cell Sci..

[37] Hung C.S., Bouckaert J., Hung D., Pinkner J., Widberg C., De Fusco A., Auguste C.G., Strouse B., Langerman S., Waksman G. (2002). Structural basis of tropism of Escherichia coli to the bladder during urinary tract infection. Mol. Microbiol..

[38] Eto D.S., Jones T.A., Sundsbak J.L., Mulvey M.A. (2007). Integrin-mediated host cell invasion by type 1-piliated uropathogenic *Escherichia coli*. PLoS Pathog..

[39] Mulvey M.A., Schilling J.D., Martinez J.J., Hultgren S.J. (2000). Bad bugs and beleagured bladders: Interplay between uropathogenic *Escherichia coli* and innate host defenses. Proc. Natl. Acad. Soc. USA.

[40] Mulvey M.A., Lopez-Boado Y.S., Wilson C.L., Roth R., Parks W.C., Heuser J., Hultgren S.J. (1998). Induction and Evasion of Host Defenses by Type 1-Piliated Uropathogenic *Escherichia coli*. Science.

[41] Martinez J.J., Mulvey M.A., Schilling J.D., Pinkner J.S., Hultgren S.J. (2000). Type 1 pilus-mediated bacterial invasion of bladder epithelial cells. EMBO.

[42] Martinez J.J., Hultgren S.J. (2002). Requirment of Rho-family GTPases in the invasion of Type-1 piliated uropathogenic *Escherichia coli*. Cell. Microbiol..

[43] Anderson G.G., Palermo J.J., Schilling J.D., Roth R., Heuser J., Hultgren S.J. (2003). Intracellular bacterial biofilm-like pods in urinary tract infections. Science.

[44] Justice S.S., Hung C., Theriot J.A., Fletcher D.A., Anderson G.G., Footer M.J., Hultgren S.J. (2004). Differentiation and developmental pathways of uropathogenic *Escherichia coli* in urinary tract pathogenesis. Proc. Natl. Acad. Soc. USA.

[45] Hung C.S., Dodson K.W., Hultgren S.J. (2009). A murine model of urinary tract infection. Nat. Protoc..

[46] Wright K.J., Seed P.C., Hultgren S.J. (2007). Development of intracellular bacterial communities of uropathogenic *Escherichia coli* depends on type 1 pili. Cell. Microbiol..

[47] Mulvey M.A., Schilling J.D., Hultgren S.J. (2001). Establishment of a persistent *Escherichia coli* reservoir during the acute phase of a bladder infection. Infect. Immun..

[48] Justice S.S., Hunstad D.A., Seed P.C., Hultgren S.J. (2006). Filamentation by *Escherichia coli* subverts innate defenses during urinary tract infection. Proc. Natl. Acad. Soc. USA.

[49] Rosen D.A., Hooton T.M., Stamm W.E., Humphrey P.A., Hultgren S.J. (2007). Detection of intracellular bacterial communities in human urinary tract infection. PLoS Med..

[50] Robino L., Scavone P., Araujo L., Algorta G., Zunino P., Vignoli R. (2013). Detection of intracellular bacterial communities in a child with *Escherichia coli* recurrent urinary tract infections. Pathog. Dis..

[51] Elliott T.S., Reed L., Slack R.C., Bishop M.C. (1985). Bacteriology and ultrastructure of the bladder in patients with urinary tract infections. J. Infect..

[52] Lim J.K., Gunther N.W., Zhao H., Johnson D.E., Keay S.K., Mobley H.L. (1998). *In vivo* phase variation of *Escherichia coli* type 1 fimbrial genes in women with urinary tract infection. Infect. Immun..

[53] Hagan E.C., Lloyd A.L., Rasko D.A., Faerber G.J., Mobley H.L. (2010). *Escherichia coli* global gene expression in urine from women with urinary tract infection. PLoS Pathog..

[54] Gunther N.W., Lockatell V., Johnson D.E., Mobley H.L. (2001). *In vivo* dynamics of type 1 fimbria regulation in uropathogenic *Escherichia coli* during experimental urinary tract infection. Infect. Immun..

[55] Hultgren S.J., Porter T.N., Schaeffer A.J., Duncan J.L. (1985). Role of type 1 pili and effects of phase variation on lower urinary tract infections produced by *Escherichia coli*. Infect. Immun..

[56] Dodson K.W., Pinkner J.S., Rose T., Magnusson G., Hultgren S.J., Waksman G. (2001). Structural basis of the interaction of the pyelonephritic *E. coli* adhesin to its human kidney receptor. Cell.

[57] Guyer D.M., Radulovic S., Jones F.E., Mobley H.L. (2002). Sat, the secreted autotransporter toxin of uropathogenic *Escherichia coli*, is a vacuolating cytotoxin for bladder and kidney epithelial cells. Infect. Immun..

[58] Mills M., Meysick K.C., O’Brien A.D. (2000). Cytotoxic necrotizing factor type 1 of uropathogenic *Escherichia coli* kills cultured human uroepithelial 5637 cells by an apoptotic mechanism. Infect. Immun..

[59] Anderson G.G., Goller C.C., Justice S., Hultgren S.J., Seed P.C. (2010). Polysaccharide capsule and sialic acid-mediated regulation promote biofilm-like intracellular bacterial communities during cystitis. Infect. Immun..

[60] Henderson J.P., Crowley J.R., Pinkner J.S., Walker J.N., Tsukayama P., Stamm W.E., Hooton T.M., Hultgren S.J. (2009). Quantitative metabolomics reveals an epigenetic blueprint for iron acquisition in uropathogenic *Escherichia coli*. PLoS Pathog..

[61] Lane M.C., Mobley H.L. (2007). Role of P-fimbrial-mediated adherence in pyelonephritis and persistence of uropathogenic *Escherichia coli* (UPEC) in the mammalian kidney. Kidney Int..

[62] Klumpp D.J., Rycyk M.T., Chen M.C., Thumbikat P., Sengupta S., Schaeffer A.J. (2006). Uropathogenic *Escherichia coli* induces extrinsic and intrinsic cascades to initiate urothelial apoptosis. Infect. Immun..

[63] Thumbikat P., Berry R.E., Zhou G., Billips B., Yaggie R., Zaichuk T., Sun T., Schaeffer A.J., Klumpp D.J. (2009). Bacteria-induced uroplakin signaling mediates bladder response to infection. PLoS Pathog..

[64] Hirose T., Kumamoto Y., Matsukawa M., Yokoo A., Satoh T., Matsuura A. (1992). Study on local immune response in *Escherichia coli*-induced experimental urinary tract infection in mice—Infiltration of Ia-positive cells, macrophages, neutrophils, T cells and B cells. Jpn. Assoc. Infect. Dis..

[65] Hopkins W.J., James L.J., Balish E., Uehling D.T. (1993). Congenital immunodeficiencies in mice increase susceptibility to urinary tract infection. J. Urol..

[66] Godaly G., Bergsten G., Frendeus B., Hang L., Hedlund M., Karpman D., Samuelsson P., Svensson M., Otto G., Wullt B. (2000). Innate defences and resistance to gram negative mucosal infection. Adv. Exp. Med. Biol..

[67] Hannan T.J., Roberts P.L., Riehl T.E., van der Post S., Binkley J.M., Schwartz D.J., Miyoshi H., Mack M., Schwendener R.A., Hooton T.M. (2014). Inhibition of Cyclooxygenase-2 Prevents Chronic and Recurrent Cystitis. EBioMedicine.

[68] Godaly G., Ambite I., Svanborg C. (2015). Innate immunity and genetic determinants of urinary tract infection susceptibility. Curr. Opin. Infect. Dis..

[69] Chen S.L., Hung C.S., Pinkner J.S., Walker J.N., Cusumano C.K., Li Z., Bouckaert J., Gordon J.I., Hultgren S.J. (2009). Positive selection identifies an *in vivo* role for FimH during urinary tract infection in addition to mannose binding. Proc. Natl. Acad. Soc. USA.

[70] Le Trong I., Aprikian P., Kidd B.A., Forero-Shelton M., Tchesnokova V., Rajagopal P., Rodriguez V., Interlandi G., Klevit R., Vogel V. (2010). Structural basis for mechanical force regulation of the adhesin FimH via finger trap-like beta sheet twisting. Cell.

[71] Aprikian P., Tchesnokova V., Kidd B., Yakovenko O., Yarov-Yarovoy V., Trinchina E., Vogel V., Thomas W., Sokurenko E. (2007). Interdomain interaction in the FimH adhesin of Escherichia coli regulates the affinity to mannose. J. Biol. Chem..

[72] Aprikian P., Interlandi G., Kidd B., Le Trong I., Tchesnokova V., Yakovenko O., Whitfield M.J., Bullitt E., Stenkamp R.E., Thomas W. (2011). The bacterial fimbrial tip acts as a mechnical force sensor. PLoS Biol..

[73] Schwartz D.J., Kalas V., Pinkner J.S., Chen S.L., Spaulding C.N., Dodson K.W., Hultgren S.J. (2013). Positively selected FimH residues enhance virulence during urinary tract infection by altering FimH conformation. Proc. Natl. Acad. Soc. USA.

[74] Schwartz D.J., Conover M.S., Hannan T.J., Hultgren S.J. (2015). Uropathogenic *Escherichia coli* superinfection enhances the severity of mouse bladder infection. PLoS Pathog..

[75] Sokurenko E.V., Vogel V., Thomas W.E. (2008). Catch-bond mechanism of force-enhanced adhesion: Counterintuitive, elusive, but... widespread?. Cell Host Microbe.

[76] Rodriquez V.B., Kidd B.A., Interlandi G., Tchesnokova V., Sokurenko E., Thomas W.E. (2013). Allosteric coupling in the bacteial adhesive protein FimH. J. Biol. Chem..

[77] Schaeffer A.J., Schwan W.R., Hultgren S.J., Duncan J.L. (1987). Relationship of type 1 pilus expression in *Escherichia coli* to ascending urinary tract infections in mice. Infect. Immun..

[78] O’Hanley P., Low D., Romero I., Lark D., Vosti K., Falkow S., Schoolnik G. (1985). Gal-Gal binding and hemolysin phenotypes and genotypes associated with uropathogenic *Escherichia coli*. N. Engl. J. Med..

[79] Wullt B., Bergsten G., Connell H., Rullano P., Gebretsadik N., Hull R., Svanborg C. (2000). P fimbriae enhance the early establishment of *Escherichia coli* in the human urinary tract. Mol. Microbiol..

[80] Zhang J.P., Normark S. (1996). Induction of gene expression in *Escherichia coli* after pilus-mediated adherence. Science.

[81] Lund B., Lindberg F., Marklund B.I., Normark S. (1987). The PapG protein is the alpha-d-galactopyranosyl-(1-4)-beta-d-galactopyranose-binding adhesin of uropathogenic *Escherichia coli*. Proc. Natl. Acad. Soc. USA.

[82] Lanne B., Olsson B.M., Jovall P.A., Angstrum J., Linder H., Marklund B.I., Bergstrum J., Karlsson K.A. (1995). Glycoconjugate receptors for P-fimbriated *Escherichia coli* in the mouse. An animal model of urinary tract infection. J. Biol. Chem..

[83] Haslam D.B., Baenziger J.U. (1996). Expression cloning of Forssman glycolipid synthetase: A novel member of the histo-blood group ABO gene family. Proc. Natl. Acad. Soc. USA.

[84] Breimer M.E., Karlsson K.A. (1983). Chemical and immunological identification of glycolipid-based blood group ABH and Lewis antigens in human kidney. Biochim. Biophys. Acta.

[85] Breimer M.E., Hansson G.C., Leffler H. (1985). The specific glycosphingolipid composition of human ureteral epithelial cells. J. Biochem..

[86] Nuccio S., Baumler A.J. (2007). Evolution of the chaperone/usher assembly pathway: Fimbrial classification goes Greek. Microbiol. Mol. Biol. Rev..

[87] Wurpel D.J., Beatson S.A., Totsika M., Petty N.K., Schembri M.A. (2013). Chaperone-usher fimbriae of *Escherichia coli*. PLoS ONE.

[88] Lillington J., Geibel S., Waksman G. (2014). Biogenesis and adhesion of type 1 and P pili. Biochim. Biophys. Acta.

[89] Parkkinen J., Rogers G.N., Korhonen T., Dahr W., Finne J. (1986). Identification of the O-linked sialyloligosaccharides of glycophorin A as the erythrocyte receptors for S-fimbriated *Escherichia coli*. Infect. Immun..

[90] Nowicki B., Selvarangan R., Nowicki S. (2001). Family of *Escherichia coli* Dr adhesins: Decay-accelerating factor receptor recognition and invasiveness. J. Infect. Dis..

[91] Goluszko P., Popov V., Selvarangan R., Nowicki S., Pham T., Nowicki B.J. (1997). Dr fimbriae operon of uropathogenic *Escherichia coli* mediate microtubule-dependent invasion to the HeLa epithelial cell line. J. Infect. Dise..

[92] Spurbeck R.R., Stapleton A.E., Johnson J.R., Walk S.T., Hooton T.M., Mobley H.L. (2011). Fimbrial Profiles Predict Virulence of Uropathogenic *E. coli* Strains: Contribution of Ygi and Yad Fimbriae. Infect. Immun..

[93] Wurpel D.J., Totsika M., Allsopp L.P., Hartley-Tassell L.E., Day C.J., Peters K.M., Sarkar S., Ulett G.C., Yang J., Tiralongo J. (2014). F9 fimbriae of uropathogenic *Escherichia coli* are expressed at low temperature and recognise Galbeta1–3GlcNAc-containing glycans. PLos ONE.

[94] Buckles E.L., Bahrani-Mougeot F.K., Molina A., Lockatell C.V., Johnson D.E., Drachenberg C.B., Burland V., Blattner F.R., Donnenberg M.S. (2004). Identification and characterization of a novel uropathogenic *Escherichia coli*-associated fimbrial gene cluster. Infect. Immun..

[95] Carey A.J., Tan C.K., Ipe D.S., Sullivan M.J., Cripps A.W., Schembri M.A., Ulett G.C. (2015). Urinary tract infection of mice to model human disease: practicalities, implications and limitations. Crit. Rev. Microbiol..

[96] Mabeck C.E. (1972). Treatment of uncomplicated urinary tract infection in non-pregnant women. Postgrad. Med. J..

[97] Ferry S., Holm S., Stenlund H., Lundholm R., Monsen T. (2004). The natural course of uncomplicated lower urinary tract infection in women illustrated by a randomized placebo controlled study. Scand. J. Infect. Dis..

[98] Hannan T.J., Mysorekar I.U., Hung C.S., Isaacson-Schmid M.L., Hultgren S.J. (2010). Early severe inflammatory responses to uropathogenic *E. coli* predispose to chronic and recurrent urinary tract infection. PLoS Pathog..

[99] Mysorekar I.U., Hultgren S.J. (2006). Mechanisms of uropathogenic *Escherichia coli* persistence and eradication from the urinary tract. Proc. Natl. Acad. Soc. USA.

[100] Schilling J.D., Hultgren S.J. (2002). Recent advances into the pathogenesis of recurrent urinary tract infections: The bladder as a reservoir for uropathogenic *Escherichia coli*. Int. J. Antimicrob. Agents.

[101] Schlager T.A., LeGallo R., Innes D., Hendley J.O., Peters C.A. (2011). B cell infiltration and lymphonodular hyperplasia in bladder submucosa of patients with persistent bacteriuria and recurrent urinary tract infections. J. Urol..

[102] Hansson S., Hanson E., Hjalmas K., Hultengren M., Jodal U., Olling S., Svanborg-Eden C. (1990). Follicular cystitis in girls with untreated asymptomatic or covert bacteriuria. J. Urol..

[103] Bleidorn J., Gagyor I., Kochen M.M., Wegscheider K., Hummers-Pradier E. (2010). Symptomatic treatment (ibuprofen) or antibiotics (ciprofloxacin) for uncomplicated urinary tract inections? Results of a randomized contolled pilot trial. BMC Med..

[104] Gagyor I., Bleidorn J., Kochen M.M., Schmiemann G., Wegscheider K., Hummers-Pradier E. (2015). Ibuprofen versus fosfomycin for uncomplicated urinary tract infection in women: randomised controlled trial. Br. Med. J..

[105] Ragnarsdottir B., Lutay N., Gronberg-Hernandez J., Koves B., Svanborg C. (2011). Genetics of innate immunity and UTI susceptibility. Nat. Rev. Urol..

[106] Arias C.A., Murray B.E. (2012). The rise of the *Enterococcus*: Beyond vancomycin resistance. Nat. Rev. Microbiol..

[107] Guiton P.S., Hung C.S., Hancock L., Caparon M.G., Hultgren S.J. (2010). Enterococcal biofilm formation and virulence in an optimized murine model of foreign body-associated urinary tract infections. Infect. Immun..

[108] Nielsen H.V., Guiton P.S., Kline K.A., Port G.C., Pinkner J.S., Neiers F., Normark S., Henriques-Normark B., Caparon M.G., Hultgren S.J. (2012). The metal ion-dependent adhesion site motif of the *Enterococcus* faecalis EbpA pilin mediates pilus function in catheter-associated urinary tract infection. mBio.

[109] Flores-Mireles A.L., Pinkner J.S., Caparon M.G., Hultgren S.J. (2014). EbpA vaccine antibodies block binding of *Enterococcus* faecalis to fibrinogen to prevent catheter-associated bladder infection in mice. Sci. Transl. Med..

[110] Singh K.V., Nallapareddy S.R., Murray B.E. (2007). Importance of the ebp (endocarditis- and biofilm-associated pilus) locus in the pathogenesis of Enteroccous faecalis ascending urinary tract infection. J. Infect. Dis..

[111] Goble N.M., Clarke T., Hammonds J.C. (1989). Histological changes in the urinary bladder secondary to urethral catheterisation. Br. J. Urol..

[112] Glahn B.E., Braendstrup O., Olesen H.P. (1988). Influence of drainage conditions on mucosal bladder damage by indwelling catheters. II. Histological study. Scand. J. Urol. Nephrol..

[113] Peychl L., Zalud R. (2008). Changes in the urinary bladder caused by short-term permanent catheter insertion. Cas. Lek. Cesk..

[114] Flores-Mireles A.L., Walker J.N., Bauman T.M., Potretzke A.M., Schreiber H.L., Park A.M., Pinkner J.S., Caparon M.G., Hultgren S.J., Desai A. (2016). Fibrinogen release and deposition on urinary catheters placed during urologic procedures. J. Urol..

[115] Rivera J., Vannakambadi G., Hook M., Speziale P. (2007). Fibrinogen-binding proteins of Gram-positive bacteria. Thromb Haemostasis..

[116] Guiton P.S., Cusumano C.K., Kline K.A., Dodson K.W., Han Z., Janetka J.W., Henderson J.P., Caparon M.G., Hultgren S.J. (2012). Combinatorial small-molecule therapy prevents uropathogenic *Escherichia coli* catheter-associated urinary tract infections in mice. Antimicrob. Agents Chemother..

[117] Alteri C.J., Hagan E.C., Sivick K.E., Smith S.N., Mobley H.L. (2009). Mucosal immunization with iron receptor antigens protects against urinary tract infection. PLoS Pathog..

[118] Roberts J.A., Kaack M.B., Baskin G., Chapman M.R., Hunstad D.A., Pinkner J.S., Hultgren S.J. (2004). Antibody responses and protection from pyelonephritis following vaccination with purified *Escherichia coli* PapDG protein. J. Urol..

[119] Billips B.K., Yaggie R.E., Cashy J.P., Schaeffer A.J., Klumpp D.J. (2009). A live-attenuated vaccine for the treatment of urinary tract infection by uropathogenic *Escherichia coli*. J. Infect. Dis..

[120] Langermann S., Palaszynski S., Barnhart M., Auguste G., Pinkner J.S., Burlein J., Barren P., Koenig S., Leath S., Jones C.H. (1997). Prevention of Mucosal *Escherichia coli* Infection by FimH-Adhesin-Based Systemic Vaccination. Science.

[121] Langermann S., Mollby R., Burlein J.E., Palaszynski S.R., Auguste C.G., DeFusco A., Strouse R., Schenerman M.A., Hultgren S.J., Pinkner J.S. (2000). Vaccination with FimH adhesin protects cynomolgus monkeys from colonization and infection by uropathogenic *Escherichia coli*. J Infect. Dis..

[122] Roberts J.A., Hardaway K., Kaack B., Fussell E.N., Baskin G. (1984). Prevention of pyelonephritis by immunizaiton with P-fimbriae. J. Urol..

[123] Goluszko P., Goluszko E., Nowicki B., Nowicki S., Popov V., Wang H.Q. (2005). Vaccination with purified Dr. Fimbriae reduces mortality associated with chronic urinary tract infection due to Escherichia coli bearing Dr. adhesin. Infect Immun..

[124] Kranjcec B., Papes D., Altarac S. (2014). D-mannose powder for prophylaxis of recurrent urinary tract infections in women: a randomized clinical trial. World J. Urol..

[125] Han Z., Pinkner J.S., Ford B., Obermann R., Nolan W., Wildman S.A., Hobbs D., Ellenberger T., Cusumano C.K., Hultgren S.J. (2010). Structure-based drug design and optimization of mannoside bacterial FimH antagonists. J. Med. Chem..

[126] Kleeb S., Pang L., Mayer K., Eris D., Sigl A., Preston R.C., Zihlmann P., Sharpe T., Jakob R.P., Abgottspon D. (2015). FimH antagonists: bioisosteres to improve the in vitro and in vivo PK/PD profile. J. Med. Chem..

[127] Cusumano C.K., Pinkner J.S., Han Z., Greene S.A., Ford B.A., Crowley J.R., Henderson J.P., Janetka J.W., Hultgren S.J. (2011). Treatment and prevention of urinary tract infection with orally active FimH inhibitors. Sci. Transl. Med..

[128] Totsika M., Kostakioti M., Hannan T.J., Upton M., Beatson S.A., Janetka J.W., Hultgren S.J., Schembri M.A. (2013). A FimH inhibitor prevents acute bladder infection and treats chronic cystitis caused by multidrug-resistant uropathogenic *Escherichia coli* ST131. J. Infect. Dis..

[129] Klein T., Abgottspon D., Wittwer M., Rabbani S., Herold J., Jiang X., Kleeb S., Luthi C., Scharenberg M., Bezenuon J. (2010). FimH antagonists for the oral treatment of urinary tract infections: From design and synthesis to *in vitro* and *in vivo* evaluation. J. Med. Chem..

[130] Bouckaert J., Berglund J., Schembri M., De Genst E., Cools L., Wuhrer M., Hung C.S., Pinkner J., Slattegard R., Zavialov A. (2005). Receptor binding studies disclose a novel class of high-affinity inhibitors of the Escherichia coli FimH adhesin. Mol. Microbiol..

[131] Aberg V., Almqvist F. (2007). Pilicides-small molecules targeting bacterial virulence. Org. Biomol. Chem..

[132] Aberg V., Fallman E., Axner O., Uhlin B.E., Hultgren S.J., Almqvist F. (2007). Pilicides regulate pili expression in *E. coli* without affecting the functional properties of the pilus rod. Mol. bioSyst..

[133] Pinkner J.S., Remaut H., Buelens F., Miller E., Aberg V., Pemberton N., Hedenstrom M., Larsson A., Seed P., Waksman G. (2006). Rationally designed small compounds inhibit pilus biogenesis in uropathogenic bacteria. Proc. Natl. Acad. Soc. USA.

[134] Piatek R., Zalewska-Piatek B., Dzierzbicka K., Makowiec S., Pilipczuk J., Szemiako K., Cyranka-Czaja A., Wojciechowski M. (2013). Pilicides inhibit the FGL chaperone/usher assisted biogenesis of the Dr fimbrial polyadhesin from uropathogenic *Escherichia coli*. BMC Microbiol..

[135] Greene S.E., Pinkner J.S., Chorell E., Dodson K.W., Shaffer C.L., Conover M.S., Livny J., Hadjifrangiskou M., Almqvist F., Hultgren S.J. (2014). Pilicide ec240 disrupts virulence circuits in uropathogenic *Escherichia coli*. mBio.

